# The contribution of spirituality and religiousness to the quality of life of cancer patients treated with radiotherapy in Ghana: a cross-sectional study

**DOI:** 10.3332/ecancer.2025.1858

**Published:** 2025-02-25

**Authors:** Joseph Daniels, Edwin Tekpertey Glover, Kofi Adesi Kyei

**Affiliations:** 1 National Centre for Radiotherapy, Oncology and Nuclear Medicine, Korle Bu Teaching Hospital, Accra, Ghana; 2 Department of Radiography, University of Ghana, Legon, Accra, Ghana; a https://orcid.org/0000-0002-1466-150X; b https://orcid.org/0000-0003-3485-5368

**Keywords:** cancer, radiotherapy, spirituality, religiousness, quality of life

## Abstract

Cancer is one of the leading causes of morbidity and mortality globally. A significant proportion of all patients with cancer require radiotherapy as part of their treatment. Cancer can be very terrifying for patients undergoing radiotherapy, especially when it undermines their ability to hope or cope. In Ghana, where religious and spiritual beliefs play a significant role in many individuals’ lives, understanding how these factors affect patients’ well-being during cancer treatment is crucial. This study examined the contribution of spirituality and religiousness to the quality of life (QoL) of cancer patients undergoing radiotherapy in Accra, Ghana. Pre-validated questionnaires were used to collect data that were analysed using the Statistical Package for the Social Sciences software (version 20). Patient interviews were also conducted and analysed thematically. Most of the respondents were female (*n* = 58, 65%), whereas 32 (35%) were male. The mean age of the respondents was 45.8 years (SD 12.9). All the participants of the study were religious and considered themselves to be either Christian (*n* = 82, 91.1%) or Muslim (*n* = 8, 8.9%). Approximately 93% of the participants relied on their spiritual beliefs to cope with cancer. Many patients reported that spirituality and religious coping are important aspects of their experience, potentially influencing their perceived QoL during radiotherapy. Most patients considered attention to spiritual concerns an important part of cancer care by doctors (88%) and nurses (85%). Five themes were identified from the analysis of the qualitative data. These themes indicated nuanced insights into how spirituality and religiousness influence the experiences of cancer patients. The findings of this study demonstrate the need to cater not only to bodily but also to emotional, social and spiritual needs that arise in the lives of cancer patients.

## Introduction

Cancer is the second leading cause of mortality globally. Approximately 19.3 million new cancer cases and nearly 10 million cancer deaths were recorded worldwide in 2020 alone. The worldwide cancer burden is projected to reach 28.4 million cases by 2040, a 40% increase from 2020 [1]. About half of all cancer patients require radiotherapy as part of their curative treatment either as definitive monotherapy or in sequential or concurrent combination with other treatment modalities such as surgery, chemotherapy, immunotherapy and targeted or hormonal therapy [2].

Cancer can be very terrifying, especially when it undermines a person’s ability to hope or cope. The diagnosis of cancer forces an individual to consider what is a plausible object of hope (e.g., cure, better quality of life (QoL) or a painless death). It also compels one to consider the deepest hope and ultimate reason for living. Additionally, it raises anxiety concerning what to expect and how to live in the present [3]. As a result of a serious illness such as cancer, patients and their caregivers may develop challenges regarding their beliefs and/or religious values, causing a great deal of spiritual pain. Spirituality, religiousness and prior life experiences may be valuable resources for coping with the physical, mental and social challenges experienced by individuals living with cancer [4].

Spirituality is present in all cultures and societies [5]. In Ghana, spirituality refers to a personal, individual connection with a higher power or the divine, encompassing beliefs, practices and experiences that give meaning to life and influence moral values and behaviors [6]. It is generally seen as a broader concept that may or may not involve formal religious practices but emphasises a personal relationship with the sacred and the quest for purpose and understanding in life. Religion, however, is a collection of beliefs, experiences and behaviors that are shared by a group of individuals and can be experienced solely and/or collectively [7]. Religiousness in Ghana is more specifically tied to organised religious practices, rituals and adherence to the doctrines of a particular faith. It often involves active participation in religious communities, such as churches, mosques or traditional religious gatherings and is expressed through regular worship, prayer and other forms of communal religious activities. Religiousness is closely linked with social identity and community belonging, reflecting the collective nature of Ghanaian society [6, 8]. In the Ghanaian context, **spirituality** and **religiousness** are deeply intertwined with cultural and communal life, though they are distinct concepts. For some people, this manifests in a yearning for substantial meaning through belief in God, rationalism, humanism and art among others [9]. An individual does not have to be a member of a religious organisation to be spiritual.

Spirituality provides a sense of peace and purpose, coupled with a sense of hope for individuals in times of distress. Proven ways of dealing with distress include reliance on personal experiences, ideas or beliefs that situate one’s fate in a larger framework, which could mean religiousness for one person but non-religious spiritual orientation for another [7]. Spirituality is an important part of many patients’ health and well-being. However, when it comes to comprehensive patient care in the oncology setting, spirituality is frequently overlooked. Oncological patients have been reported to have a high level of spirituality/religiosity that correlates with their physical, emotional and social functioning [10]. Similar studies conducted in other African contexts, such as Nigeria and South Africa, have shown that spirituality and religiousness significantly influence the QoL of cancer patients. For instance, research in Nigeria by Adejumo *et al* [11], highlighted that spiritual beliefs and religious practices are central to how patients cope with cancer diagnoses, with many viewing their illness as a spiritual journey. Additionally, studies in Kenya and South Africa emphasised the role of religious communities and spiritual practices in providing emotional and social support, underscoring the importance of incorporating spiritual care into cancer treatment in African settings. A study by Ngure *et al* [12] revealed that patients with cancer in Kenya often rely on spiritual practices, such as prayer and participation in religious rituals, to find hope and meaning during their cancer journey. Peltzer *et al* [13] reported that, in South Africa, oncology patients often seek comfort in their faith and believe that divine intervention plays a role in their healing process. Many individuals see terminal illness as a chance to grow spiritually [14]. In a study involving breast cancer patients, individuals with higher self-reported spirituality reported more hope and higher levels of QoL [15]. Similarly, another study evaluating the association between the spirituality and QoL of women with breast cancer undergoing radiotherapy in Brazil demonstrated a positive correlation between the ‘overall QoL score’ and multiple facets of spirituality [16]. Patients undergoing radiotherapy often rely on religious and spiritual beliefs [17].

In Ghana, where religious and spiritual beliefs play a significant role in many individuals’ lives, understanding how these factors affect patients’ well-being during cancer treatment is crucial. Despite the significance of spirituality and religiousness in African cancer care, there is a paucity of published literature on their contribution to the QoL of Ghanaian cancer patients undergoing radiotherapy. Although previous studies have explored cancer patients’ experiences in African contexts, none have specifically focused on radiotherapy patients in Ghana. Hence, this cross-sectional study explored the impact of spirituality and religiousness on the QoL of cancer patients who were undergoing radiation treatment at a prominent radiotherapy centre in Ghana, West Africa. The study provides insights into the psychological and emotional support that spirituality and religiousness offer, ultimately aiming to improve holistic cancer care and patient outcomes in the Ghanaian context.

## Methods

### Study design and setting

This research was a single-institution-based mixed method descriptive cross-sectional study. The study was conducted at a major radiation treatment centre in West Africa located within the third largest hospital in Africa. The radiotherapy centre operates a 1.25 MeV cobalt-60 teletherapy machine and a 6 MV linear accelerator, both of which are used to treat patients with cancers of various primary and metastatic sites either curatively or palliatively. The study centre is one of two publicly-funded radiotherapy centres in Ghana that receives patient referrals from all over the country as well as some neighboring sub-Saharan African countries.

### Participants

All adult patients (≥ 18 years) with a histopathologically confirmed diagnosis of cancer of any primary site undergoing radiotherapy at the study centre during the sampling period were invited to participate in the study. Participants were recruited regardless of gender, religious affiliation and spiritual beliefs. The study included patients treated with both palliative and curative intent. Patients undergoing brachytherapy and those receiving external beam radiotherapy for benign conditions or hematological malignancies such as plasmacytoma and multiple myeloma were not included in the study. Patients who were enrolled in other studies that could interfere with or confound the results of this study were also excluded. Additionally, critically ill patients were also excluded as well as those who declined or could not provide written informed consent.

### Data sources

A structured questionnaire with closed-ended questions and semi-structured interview guide were used for data collection. Quantitative data were elicited through the questionnaire whereas qualitative data were obtained by means of several in-person interviews with the participants of the study. The questionnaire consisted of two sections. The first section collected demographic information and details about the clinical diagnosis of the respondents. The second section incorporated questions addressing the research objectives. The English version of the Herth Hope Index (HHI) was used to measure the hope of respondents along three dimensions: temporality and future, positive readiness and expectancy and interconnectedness [18]. This is a 12-item self-reporting tool that uses a 4-pount Likert scale ranging from 1 (strongly disagree) to 4 (strongly agree). The cumulative score ranged from 12 to 48 with higher scores denoting higher levels of hope. The validity and reliability of this research instrument have been proven to be satisfactory [19]. The in-depth interviews thoroughly explored the contribution of spirituality and religiousness to the QoL of the participants. The participants were initially asked to describe themselves in terms of religiousness and spirituality, and subsequently, the role spirituality and religiousness play in their lives regarding their cancer diagnosis as well as how this affects their QoL. All interviews were conducted by the principal investigator, lasting between 15 and 25 minutes per interview. Pilot testing of the interview guide was conducted to ensure its suitability for eliciting the required information.

### Bias

The questionnaires were administered in English; however, not all the participants could read and understand English efficiently. A well-trained member of the research team translated the text for patients who were more comfortable with their indigenous language. Patients’ care-givers were prevented from interacting with the patients during the time of completion of the questionnaires in order not to influence the responses of the patients. They were equally prevented from completing the questionnaires on behalf of the patients who required assistance.

### Study size and sampling method

Participants were recruited for the study using a purposive sampling method. A total of 145 individual patients were treated at the study site during the sampling period, all of whom were screened for eligibility. Questionnaires were administered to all 101 eligible patients out of which 90 (89%) were appropriately completed and returned. For the qualitative aspect of the study, data saturation was detected after interviewing 60 participants (66.7%)

indicating that further data collection would no longer contribute to a better understanding of the research questions. Achieving data saturation ensured that the qualitative findings of the study were comprehensive and that the number of participants interviewed was sufficient to support the research conclusions [20, 21]. The limited number of patients interviewed reduced the risk of over-representing a specific demographic of cancer patients in this study.

### Statistical methods

Quantitative data were entered into a database and summarised using both Microsoft Excel 2019 and Statistical Package for Social Sciences version 20. Normally distributed data were presented as means with standard deviations. Patient baseline characteristics were presented in tables with frequencies and proportions. For the qualitative data, all interviews were digitally recorded with the consent of each participant and transcribed verbatim. A thematic analysis approach was employed, as proposed by Braun and Clarke [22] beginning with open coding, where key concepts and patterns within the data were identified. These codes were systematically categorised and grouped into broader themes that represented the role of spirituality and religiousness in the patients’ lives and their impact on QoL. The context of these themes was explored, ensuring that both common and unique responses were captured and analysed.

The credibility of the qualitative aspect of this study was enhanced through several strategies, including prolonged engagement, member checking, triangulation and peer debriefing. Credibility was further reinforced by deriving the research findings directly from the original data collected from participants [23]. Peer debriefing involved an independent peer with relevant expertise who was not directly involved in the study, and who reviewed the research process comprehensively. This review covered the study design, data collection methods, data analysis and research findings.

### Ethical considerations

Ethical approval was obtained from the institutional review board before the commencement of the study. Written informed consent was obtained from the participants after the aim of the study had been thoroughly explained in a language they could understand. The anonymity and confidentiality of patients were always ensured during the study. All data collected were used for the purpose of this study only.

## Results

### Baseline characteristics of participants

**Figure 1.**
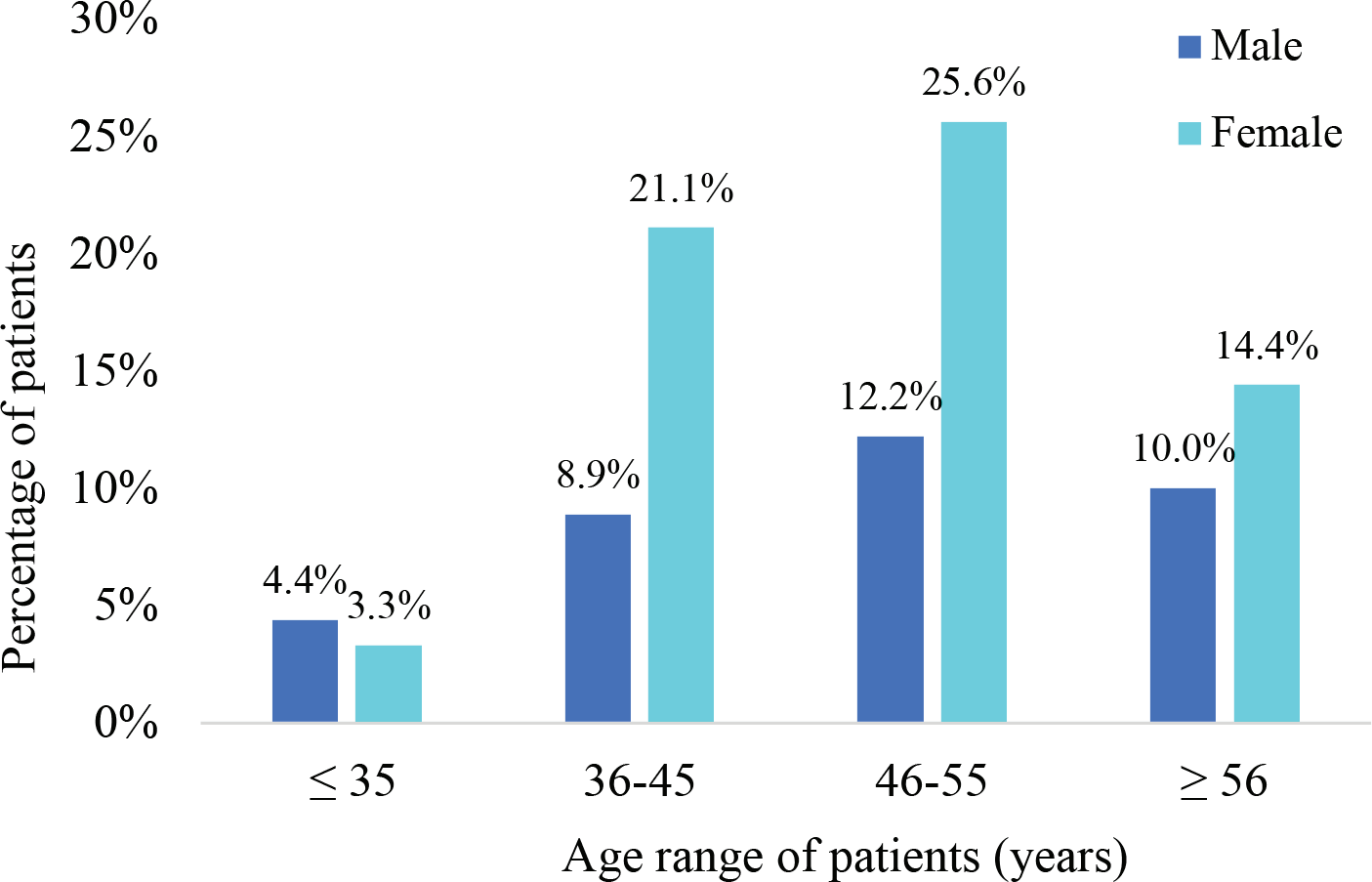
The age distribution of the participants based on gender.

The study population encompassed all adult cancer patients undergoing radiotherapy at the study site. Questionnaires were given out to a total of 101 eligible patients at a response rate of 89.1%. Most of the respondents were female (*n* = 58, 65%) whereas 32 (35%) were male. The mean age of the respondents was 45.8 years (SD 12.9). The youngest patient was 28 years whereas the oldest was 65 years. There were more males than females among the patients who were ≤35 years; however, there were significantly more females than males in all other age categories as illustrated in Figure 1.

Most of the respondents (*n* = 48, 53.3%) were married whereas the remainder were either single, divorced or widowed as shown in Table 1 which summarises the baseline and treatment-related characteristics of the patients who participated in this study. In all, 67 (74.5%) of the patients were employed at the time of undergoing radiotherapy whereas the others were unemployed. A total of 21 (23%) respondents had received no formal education at all whereas the others had received formal education at the elementary, secondary and tertiary levels. In all, 39 (43.3%) of the patients were receiving treatment for breast cancer whereas 10 (11.1%), 17 (18.8%) and 9 (10%) were treated for cervical, prostate and head and neck cancers, respectively. Most of the patients were treated with curative intent (*n* = 76, 84.4%) and on the cobalt-60 teletherapy machine (*n* = 63, 70%).

**Figure 2.**
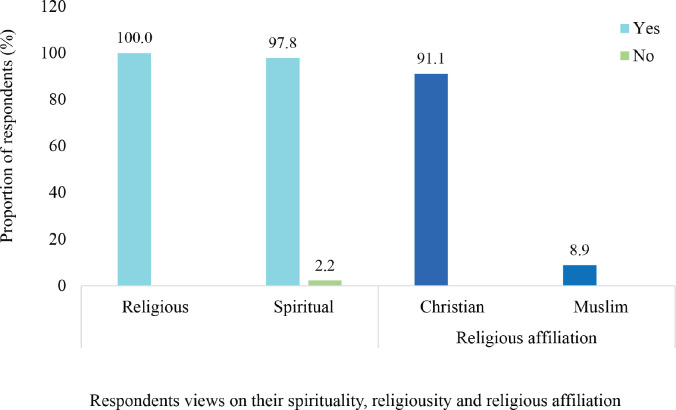
Distribution of respondents based on their spirituality, religiousness and religious affiliation. This summarises the views of the respondents about their individual spirituality and religiosity. These views were expressed in answer to the question ‘Do you consider yourself to be spiritual and/or religious?’. The proportion of respondents who belong to an identifiable religious group is also demonstrated. All the respondents considered themselves either Christian or Muslim.

All the participants of the study were religious and considered themselves to be either Christian (*n* = 82, 91.1%) or Muslim (*n* = 8, 8.9%). On the other hand, 88 (91.1%) of the respondents considered themselves to be spiritual whereas 2 (2.2%) were not spiritual as shown in Figure 2 which illustrates the distribution of the views of the respondents based on their spirituality, religiousness and religious affiliation.

### Contribution of spirituality and religiousness to patients’ QoL

Table 2 summarises the views of the respondents concerning the extent of their spirituality and religiousness as well as how these influence their QoL and help them cope with the diagnosis of cancer. The majority of the patients considered themselves to be extremely spiritual (*n* = 48, 53.3%) and extremely religious (*n* = 42, 46.7%). Even though all the respondents considered themselves to be religious to some extent, not all of them identified themselves as being spiritual. Only 2(2.3%) were religious but not simultaneously spiritual. A total of 63 respondents (69.2%) viewed spirituality as an object of hope. Out of all the respondents, 76 (84.4%) acknowledged that spirituality and religiousness assist them to various extents in dealing with cancer whereas 14 (15.6%) denied any such role of spirituality and religiousness in their lives regarding how they cope with cancer. Similarly, 82 (91.1%) of the patients being treated for cancer confirmed the contribution of both spirituality and religiousness to their QoL. However, 8 (8.9%) of the respondents denied any positive contribution of spirituality and religiousness to their QoL.

### Perception of spiritual care

Items 4 and 11 of the HHI were the most strongly agreed to by the participants. Also, none of the respondents strongly disagreed with items 4, 5, 10, 11 and 12. A summary of the patients’ perception of spiritual care based on the HHI is shown in Table 3.

Table 4 summarises the mean scores of the respondents of the study on the HHI. The highest mean score (3.48, SD 1.03) was recorded for the item on ‘seeing possibilities amid difficulties’ whereas the lowest score (2.33, SD 0.29) was recorded for the item on ‘feeling alone’. ‘Giving and receiving care/love’ and ‘each day having a potential’ also had high mean scores of 3.42 (SD 0.96) and 3.40 (0.96), respectively.

### Themes

Five themes were identified from the analysis of the qualitative data, namely, ‘religious teachings provide assurance of hope for the future’, ‘there is comfort and reassurance when the doctors and nurses taking care of patients appreciate the spirituality and/or religiousness of patients’, ‘religious and spiritual patients believe in and anticipate supernatural healing’, ‘there is increased anxiety and stress when patients’ clinical conditions advance despite their adherence to spiritual and religious beliefs’, and ‘patients often seek spiritual/religious counsel before commencing treatment when diagnosed with cancer’.

#### Theme #1: Religious teachings provide assurance of hope for the future

For several patients with cancer, religious teachings serve as a source of hope and reassurance about their future. These teachings often emphasise the possibility of a better outcome, whether in this life or in the afterlife. The belief in a higher power’s plan and the idea that their suffering has a purpose helps patients maintain a positive outlook despite the challenges they face during their treatment.

Interview excerpts:

*“My pastor always reminds me that God has a plan for me, and that gives me the strength to face each day, no matter how difficult the treatment gets.”*

*“My faith teaches me that there’s always hope, even when the doctors don’t give me much of it. That hope is what keeps me going.”*

*“Whenever I feel down, I remember the teachings that God is in control of everything, and that alone brings me peace and hope.”*

#### Theme #2: There is comfort and reassurance when the doctors and nurses taking care of patients appreciate the spirituality and/or religiousness of their patients

Some patients feel more comfortable and reassured when healthcare providers acknowledge and respect their spiritual and religious beliefs. This recognition helps to build trust between the patients and their caregivers, making patients feel that their care is holistic and that their spiritual needs are being considered alongside their physical treatment.

Interview excerpts:

*“It means a lot to me when the nurses and radiation therapists ask if they can pray with me before starting my treatment. It shows that they care about more than just my physical condition.”*

*“When the doctors (radiation oncologists) demonstrate respect for my religious practices, like praying and reading “healing psalms” before treatment, it makes me feel more confident that I’m in good hands.”*

*“I feel so much at peace knowing that my doctor understands my faith and encourages me to lean on it during this difficult time (time of cancer treatment).”*

*“The comfort I get when my caregivers acknowledge my spirituality is immense. It feels like they are taking care of my soul as well as my body.”*

*“The nurses (patient navigators) frequently ask how my faith is helping me cope. I am comforted, knowing that they see my spiritual well-being as part of my overall care.”*

#### Theme #3: Religious and spiritual patients believe in and anticipate supernatural healing

Many cancer patients with strong religious and spiritual beliefs hold on to the hope of supernatural healing. This belief often coexists with their medical treatment, where they anticipate that their faith and prayers could lead to a miraculous recovery. Such beliefs can provide significant psychological comfort and a sense of control over their long-term survival outcomes.

Interview excerpts:

*“I believe that God can heal me completely, even when the doctors say the (prostate) cancer is advanced and incurable. My faith tells me that, with God nothing is impossible.”*

*“I seek medical treatment for my (breast) cancer, but I also trust in divine healing. I believe that through prayer and fasting, I will experience supernatural healing.”*

*“My pastor told me to have faith in God and believe in a miracle. I am expecting that miracle every day. I have seen other people in my church with all kinds of medical conditions healed by prayer, and I believe the same will happen for me.”*

*“I’m not just relying on radiotherapy for complete healing; I’m also praying for a miracle. I believe that God will heal me in His time.”*

*“Being in church for so many years has taught me to believe in miracles and expect the “humanly impossible”. I believe that my cancer will be cured by the time I complete radiotherapy even though the doctors told me the purpose of the treatment was just for local control because the cancer (cervical cancer) has already spread to the liver “.*

#### Theme #4: There is increased anxiety and stress when patients’ clinical conditions advance despite their adherence to spiritual and religious beliefs

Patients diagnosed with cancer who strongly adhere to their spiritual and religious beliefs may experience increased anxiety and stress when their clinical condition worsens, despite their faith and prayers. This can lead to feelings of confusion, doubt and spiritual distress, as they struggle to reconcile their faith, spiritual expectations and religious beliefs with their deteriorating health.

Interview excerpts:

*“I have been praying and fasting since my doctor told me I have an advanced cancer of the cervix, but my condition is still getting worse. I am anxious about where all this is headed and question why this is happening to me.”*

*“I expected my faith to protect me from the cancer spreading, but now that it has, I’m distressed and worried about what will happen to me next.”*

*“My belief was supposed to give me peace, but now I’m just scared because I don’t understand why my condition isn’t improving. At first, I was very calm and hopeful about my diagnosis but now I am very worried and can barely even sleep because it seems my prayers are not working. Sometimes, I wonder if I have done something wrong.”*

#### Theme #5: Patients often seek spiritual/religious counsel before commencing treatment when diagnosed with cancer

Many patients seek spiritual/religious guidance before starting their cancer treatment. This counsel provides them with emotional and psychological support, helping them to prepare mentally and spiritually for the challenges ahead. Such guidance often reinforces their faith and gives them the strength to face their treatment with a positive mindset.

Interview excerpts:

*“Before starting radiotherapy, I went to see my pastor. His prayers and counsel gave me the courage to undergo the treatment despite the side effects I was told about by the doctors.”*

*“I had to talk to my spiritual father (leader) first, before accepting the treatment regimen recommended by my doctor. His (the spiritual leader) advice and blessings were crucial in order for me to feel ready to begin treatment.”*

*“I consulted with my religious mentor before agreeing to the treatment plan. It was important for me to have that spiritual go-ahead (approval).”*

*“My family and I prayed together to seek the will of God before I started my treatment. It was a necessary step for me to feel at peace and to be assured I was making the right decision. I wouldn’t have started the treatment otherwise”.*

## Discussion

All the patients considered themselves to be religious whereas only 97.8% considered themselves to be spiritual. However, all the patients were affiliated with a religious group; either Christianity or Islam. A significant proportion of the respondents were extremely spiritual (53.3%) and extremely religious (46.7%). Spirituality was an object of hope for 69.2% of the patients. Spirituality and religiousness were reported to assist most of the patients (84.4%) in dealing with cancer to various extents. For 18.9% they were the most important things that kept them going whereas for 15.6% both played no role at all in how the patients deal with cancer. Spirituality and religiousness were also reported to contribute to the QoL of 91.1% of the patients albeit to different extents. On the other hand, for 8.9% of the patients, neither spirituality nor religiousness contributed to their QoL. Items with the highest mean scores on the HHI were ‘seeing possibilities amid difficulties’ (3.48, SD 1.03), ‘giving and receiving care/love’ (3.42, SD 0.96) and ‘each day having its potential’ (3.4, SD 0.83). On the other hand, items with the lowest scores were ‘presence of short and/or long-term goals’ (2.81, SD 0.63), ‘feel scared about my future’ (2.26, SD 0.37) and ‘feeling alone’ (2.33, SD 0.29).

Spirituality is a personal affair and a crucial component of many cancer patients’ coping strategies. Most of the cancer patients receiving radiation therapy in this study considered themselves religious and/or spiritual and relied on this as a coping mechanism for dealing with their cancer diagnosis. Interestingly, although all the patients were affiliated with one religious group or another, not all considered themselves to be spiritual. Thus, signifying that mere religious affiliation does not necessarily imply spirituality even though the two are intricately linked. Similarly, the patients who were extremely religious were not necessarily extremely spiritual. Individuals usually become affiliated with a particular religious group based on their family background and the primary religious affiliation of their immediate family members. To some extent, religious affiliation promotes social acceptance which comes in handy for cancer patients.

Some patients consider cancer to be a spiritual disease that must be dealt with spiritually. For these patients, it is understandable why they would be extremely religious when faced with the diagnosis and prognosis of cancer. There is anecdotal evidence of some cancer patients presenting late for medical treatment due to prolonged periods of time spent pursuing religious and/or spiritual remedies. For this reason, it remains essential for stakeholders in the healthcare industry to engage with religious leaders to promote early reporting of symptoms of disease at healthcare facilities. The complementarity of modern (western) medicine and religious or spiritual remedies to illness must be comprehensively addressed.

For 15.6% of the patients, religiousness and spirituality had no role in their coping mechanisms for dealing with cancer. Even though all the patients were affiliated with one religion or the other, approximately 91% considered themselves to be spiritual. This attests to the fact that religiousness and spirituality play different roles in the lives of cancer patients. On one hand, they can provide a basis for hope and optimism whereas, on the other hand, they can generate thoughts of disappointment, despair and rejection by God. Even though religiousness and spirituality provide comfort, meaning and hope, they may also be harmful if patients perceive their illness as a punishment or if God is seen as weak, distant or uncaring [24]. In a study evaluating the association between religiosity, depression and anxiety among Moroccan cancer patients, it was demonstrated that engaging in religious practices significantly decreased the risk of suffering from depression and/or anxiety. However, some religious and/or spiritual practices were also shown to have the opposite effect [25]. Interestingly, a small study involving 39 Lebanese cancer patients did not find any relationship between spirituality and either depression or QoL. After adjusting for depression, the researchers found a negative association between spirituality and QoL. The authors opined that the cumulative physical and psychological challenges faced by patients with chronic illnesses may overshadow and diminish any potential influence of spirituality on their QoL [26].

The results of this study indicate that for a lot of cancer patients, the acknowledgement of their religiousness and/or spirituality by their care givers is an essential part of their overall cancer treatment. During the interviews, some patients indicated that doctors and nurses were not always accommodating of patients’ spirituality and religious beliefs. Some patients expressed the need for their healthcare providers to consistently acknowledge and accommodate the religious beliefs and spirituality of patients. Healthcare providers must intentionally make provisions for the religious and spiritual care of cancer patients. In some health facilities, there are designated prayer rooms where patients can worship and engage in other religious activities as in-patients. Other healthcare providers also engage the services of religious leaders such as priests, chaplains, pastors or Imams who offer religious services to cancer patients. The rights, wishes and desires of cancer patients who are not open to such services must equally be respected.

In this study, the connection between spirituality and improved QoL in patients undergoing radiotherapy is similar to that seen in previous studies showing the importance of religiousness and spirituality to patient wellness in other populations facing terminal illnesses. In a study of 44 possible predictors of patient QoL as one is nearing death (e.g., death at home), patients consistently rated being ‘at peace with God’ and ‘free from pain’ as the two most significant factors affecting their well-being at the final stages of life. Notably, this study demonstrates that spirituality is related to significantly better QoL amid a high level of physical stress. The intricate association between religiousness/spirituality and patients’ QoL is independent of their socioeconomic and cancer-related clinical behaviors and conditions [27]. It is reported that incorporating spiritual care into cancer care improves key outcomes for patients [28]. At the 3-week follow-up point in a study that randomly assigned patients to spiritual evaluation by healthcare givers versus usual care, cancer patients having spirituality assessments had lower depressive symptoms and higher QoL compared to the control patients. Patients who received the intervention were also more content with their doctor’s care and gave the professional better reviews for compassion, gentleness, respect and patient-physician communication. In a similar fashion, attending to patients’ spiritual issues by the healthcare personnel was linked to better patient QoL and less hostile medical treatment close to death [29]. A meta-analysis on the influence of Islamic religion and spirituality on the well-being and QoL of cancer patients showed that religion- or spirituality-based interventions improve the well-being/QoL of cancer patients [30]. These findings emphasise the importance of all oncology practitioners taking spirituality into account in cancer patients as part of comprehensive cancer care [31]. Clinicians play a central role in addressing spiritual concerns, referring patients to chaplaincy and/or spiritual advocates in society, and possibly engaging in a spiritual practice such as patient-practitioner prayer. Evidence-based recommendations for addressing spirituality in serious illnesses like cancer include incorporating patient-centred and evidenced-based approaches in patient care, increasing awareness among health professionals, and recognising spirituality as a social determinant of health [32].

The HHI has been used to assess hope and its impact on the QoL to show that patients with higher levels of hope had better QoL and were better able to cope with their illness. The patients who scored higher on the HHI had better coping skills and were more likely to seek help when facing stressful situations [33]. Another study showed that those who scored higher on the HHI had greater levels of resilience, better coping skills and greater satisfaction with life [34]. The originator of the hope index used in this study defines hope as ‘a multidimensional life force characterised by a confident yet uncertain expectation of achieving a future good which the hoping person considers to be personally significant’ [18]. A study among Jordanian patients with breast cancer reported a positive linear relationship between patients’ spirituality and their QoL [35]. The spirituality and religiousness of cancer patients are important dimensions to take into consideration when caring for cancer patients to optimise their QoL [10]. This can be especially very important for patients with advanced, metastatic or incurable disease.

The qualitative findings from the study reveal the profound impact of spirituality and religiousness on the experiences of cancer patients undergoing radiotherapy in Ghana. Patients often draw inspiration from religious teachings that emphasise hope and optimism. These teachings, which focus on divine plans and the promise of a better afterlife, help patients maintain resilience in the face of difficulties emanating from their cancer diagnosis and treatment [36, 37]. For many patients, the belief in supernatural healing through faith and prayer coexists with conventional medical treatment. This belief serves as a psychological anchor, offering patients hope for a miraculous recovery, even in cases where medical prognosis is poor [38, 39]. The anticipation of divine intervention provides a sense of control over their illness, which can be empowering for patients who might otherwise feel helpless [40]. This expectation of supernatural healing, while not a replacement for medical treatment, can boost patients’ morale and contribute to their overall sense of well-being during the treatment process [41]. It reflects the deep intertwining of faith and medicine in the patients’ coping strategies. When patients’ health deteriorates despite their adherence to spiritual practices and beliefs, it can lead to significant anxiety and spiritual distress. This theme highlights the internal conflict that arises when the anticipated divine intervention does not manifest as expected. The failure to reconcile their spiritual expectations with their clinical reality can leave patients feeling isolated and vulnerable, underscoring the need for compassionate spiritual support during such times [42]. Before commencing cancer treatment, many patients seek guidance from spiritual or religious leaders, which plays a critical role in their decision-making process. This counsel helps them mentally and emotionally prepare for the challenges ahead, providing a sense of reassurance and divine approval [43]. By aligning their treatment with their spiritual beliefs, patients feel more confident and supported as they embark on their medical journey. This step often serves as a foundation for their coping mechanisms, helping them face the physical and emotional demands of cancer treatment with a fortified sense of purpose and inner peace [39, 43]. It also illustrates the deep integration of faith in the lives of these patients, where spiritual counsel is seen as essential to making informed and meaningful decisions about their health [36].

## Limitations

Data collected for this study were based on participants’ self-reported experiences, which could have been influenced by social desirability bias, memory recall issues or discomfort in discussing sensitive topics. The study reports a Christian-to-Muslim ratio of approximately 10:1 in terms of religious affiliation among cancer patients receiving radiation therapy. This ratio does not reflect the proportion of cancer patients affiliated with either group in the general population. Additionally, even though the patients in this study were affiliated with only two popular religious groups, it would be inaccurate to infer that there are no cancer patients who are affiliated with other religious worldviews (such as Hinduism, Shintoism, Buddhism and so on) or cancer patients who do not associate themselves with any religious groups at all. A rerun of the methodology at a different cancer site may produce very different results regarding the abovementioned patient-related characteristics. The use of thematic analysis also introduces potential limitations, including subjectivity in coding and loss of nuance from verbal to written translation, which may affect the depth of the findings and generalisability of the central conclusions. Hence, the findings of this study should be carefully interpreted bearing the above-mentioned limitations in mind.

## Recommendations

Healthcare providers should integrate spiritual care into oncology practice by training staff to engage with and respect patients’ spiritual and religious beliefs, enhancing patient comfort and trust. A standardised spiritual assessment should be conducted to understand patients’ beliefs, with spiritual support provided based on their needs. Establishing dedicated support systems, such as spiritual counseling services, is crucial for addressing spiritual distress, particularly for patients experiencing anxiety due to deteriorating health despite their faith. Open communication between patients and healthcare providers regarding spiritual beliefs should be encouraged to align care with patients’ values. Further research should explore the psychological impact of spirituality and beliefs in supernatural healing, which are central to patients’ coping mechanisms, to design interventions that improve QoL without compromising medical treatment. Additionally, studies should assess the availability and accessibility of spiritual services at cancer treatment centres in Ghana. Further research should also be conducted to investigate the impact of religious coping on cancer patients affiliated with other religious groups in Ghana to better understand how to assist them.

## Conclusion

Spirituality and religiousness play an important role in the lives of cancer patients who undergo radiation treatment in low-resource settings such as Ghana. These two factors directly affect patients’ QoL and should be routinely assessed for all patients. Spiritual care should be incorporated into the comprehensive care of cancer patients. The findings of this study demonstrate the need to cater not only to bodily but also to emotional, social and spiritual needs that arise in the lives of cancer patients. This study adds new insights to the existing body of scientific literature on the important contribution of spirituality and religion to the QoL of patients diagnosed with cancer in the Ghanaian setting.

## Conflicts of interest

The authors declare that they have no conflicts of interest.

## Funding

The authors declare that no funds, grants, awards or other support were received either during the conduct of the study or during the preparation of the manuscript.

## Author contributions

**Joseph Daniels:** Conceptualisation, Methodology, Data curation, Formal analysis, Visualisation, Supervision, writing – Reviewing and editing. **Edwin Tekpertey Glover:** Methodology, Formal Analysis, Investigation, Writing – Original draft. **Kofi Adesi Kyei:** Resources, Validation, Writing – Reviewing and editing, Visualisation, Supervision.

## Figures and Tables

**Table 1.** Sociodemographic and treatment-related characteristics of the respondents.

Characteristics	Variables	Number (*N* = 90)	Percentage (%)
Sex	Male	32	35
	Female	58	65
Marital status	Single	12	13.3
	Married	48	53.3
	Divorced	11	12.2
	Widowed	19	21.1
Employment status	Employed	67	74.5
	Unemployed	23	25.5
Educational level	Elementary	18	20
	Secondary	30	34
	Tertiary	21	23
	None*	21	23
Site of primary cancer	Breast	39	43.3
	Cervix	10	11.1
	Prostate	17	18.8
	Head & neck	9	10
	Other	15	16.8
Treatment machine	Cobalt-60	63	70
	6 MV Linac	27	30
Treatment intent	Curative	76	84.4
	Palliative	14	15.6

MV: megavolts. The table summarises the sociodemographic characteristics of the respondents of the study as well as characteristics of their treatment including the primary cancer site, intent of treatment and the teletherapy machine used for treatment. *These were patients who received no formal education

**Table 2.** Summary of patients’ responses to questions about the extent of their spirituality/religiousness and the contribution to their QoL.

Characteristics	Variables	Number (*N* = 90)	Percentage (%)
Extent of spirituality	Extremely spiritual	48	53.3
	Moderately spiritual	31	34.4
	Slightly spiritual	9	10
	Not spiritual at all	2	2.3
Extent of religiousness	Extremely religious	42	46.7
	Moderately religious	35	38.9
	Slightly religious	13	14.4
	Not religious at all	0	0
Spirituality as an object of hope	Yes	63	69.2
	No	27	30.8
Extent to which spirituality and religiousness assist patients in dealing with cancer	It is the most important thing that keeps me going	17	18.9
	To a large extent	30	33.3
	To a moderate extent	23	25.6
	To a small extent	6	6.7
	Not at all	14	15.6
Extent to which spirituality and religiousness contribute positively to the QoL of patients	It is the most important thing that keeps me going	30	33.3
	To a large extent	15	16.7
	To a moderate extent	25	27.8
	To a small extent	12	13.3
	Not at all	8	8.9

This is a summary of the extent to which the respondents considered themselves either religious and/or spiritual. The table also summarises the views of respondents on the impact of spirituality on their QoL and the extent to which it helps them cope with the diagnosis of cancer.

**Table 3.** Summary of the participants’ perception of spiritual care based on the HHI (*N* = 90).

Questionnaire item	Strongly disagree	Disagree	Agree	Strongly agree
I have a positive outlook toward life	13	8	26	43
I have short and/or long-term goals	4	26	45	15
I feel all alone	33	15	21	21
I can see possibilities amid difficulties	0	8	33	49
I have a faith that gives me comfort	0	19	28	43
I feel scared about my future	40	10	16	24
I can recall happy/joyful times	1	1	55	33
I have deep inner strength	1	9	40	40
I can give and receive caring/love	1	7	39	43
I have a sense of direction	0	20	34	36
I believe each day has its potential	0	15	28	47
I feel my life has value and worth	0	5	44	31

The table shows the number of patients who either strongly disagreed, disagreed, agreed or strongly agreed with each of the 12 items on the HHI.

**Table 4.** Mean item scores of the respondents on the HHI.

Individual items	Mean (SD)
1. Positive outlook toward life	3.15 (0.87)
2. Presence of short and/or long-term goals*	2.81 (0.63)
3. Feel all alone	2.33 (0.29)
4. See possibilities amid difficulties*	3.48 (1.03)
5. Faith that comforts*	3.30 (0.86)
6. Feel scared about my future	2.26 (0.37)
7. Recall happy/joyful times	3.32 (0.95)
8. Deep inner strength	3.36 (0.90)
9. Give and receive caring/love	3.42 (0.96)
10. Sense of direction	3.20 (0.73)
11. Each day has its potential	3.40 (0.96)
12. Feel my life has value and worth	2.95 (0.83)

* Items 2,4 and 5 are reworded (the original version of item 2 = I have short, intermediate and/or long-range goals, the original version of item 4 = I can see a light in the tunnel, the original version of item 5 = I have faith that gives me comfort).
